# How to study the impact of sex and gender in medical research: a review of resources

**DOI:** 10.1186/s13293-016-0099-1

**Published:** 2016-10-14

**Authors:** Alyson J. McGregor, Memoona Hasnain, Kathryn Sandberg, Mary F. Morrison, Michelle Berlin, Justina Trott

**Affiliations:** 1 Division of Sex and Gender in Emergency Medicine, Department of Emergency Medicine, Alpert Medical School of Brown University, Providence, RI USA; 2 Department of Family Medicine, College of Medicine, University of Illinois at Chicago, Chicago, IL USA; 3 Center for the Study of Sex Differences in Health, Aging and Disease, Georgetown University Medical Center, Washington, DC, USA; 4 Department of Psychiatry, Temple University School of Medicine, Philadelphia, PA USA; 5 Department of Internal Medicine, Temple University School of Medicine, Philadelphia, PA USA; 6 Center for Women’s Health, Oregon Health & Science University, Portland, OR USA; 7 University of New Mexico, Albuquerque, NM USA

**Keywords:** Sex and gender, Resource, Research methods, Sex analysis, Gender analysis

## Abstract

There is a growing appreciation by the biomedical community that studying the impact of sex and gender on health, aging, and disease will lead to improvements in human health. Sex- and gender-based comparisons can inform research on disease mechanisms and the development of new therapeutics as well as enhance scientific rigor and reproducibility. This review will assist basic researchers, clinical investigators, as well as epidemiologists, population, and social scientists by providing an annotated bibliography of currently available resource tools on how to consider sex and gender as independent variables in research design and methodology. These resources will assist investigators applying for funding from the National Institutes of Health since all grant applicants will be required (as of January 25, 2016) to address the role of sex as a biological variable in vertebrate animal and human studies.

## Background

While sex and gender differences in the etiology, age of disease onset, symptomology, diagnostics, response to treatment, and outcomes have long been known to exist for key health outcomes in medicine, insufficient biomedical research and reporting on the impact of sex and gender in health and disease still exists. For instance, sex and gender differences exist in the pharmacokinetics and pharmacodynamics of drug action [1]; however, most basic science research is conducted based on a male model. A survey of ten different disciplines including neuroscience, physiology, pharmacology, endocrinology, zoology, and behavioral science demonstrated that the majority of studies published in 2009 were conducted in male animals [2]. Furthermore, the Food and Drug Administration (FDA) does not require phase II clinical studies to compare dose and efficacy between men and women. Women made up less than 33 % of the participants in phase I trials approved by the FDA between 2006 and 2007 [3], and women have been underrepresented in phase III clinical trials of certain drug classes, including those that target renal and cardiovascular disease [4]. Moreover, there are no mandatory FDA requirements for prospectively designing clinical trials to investigate the impact of one’s sex on drug receptivity or adverse effects or for conducting appropriate and complete analyses by sex.

To address the underrepresentation of females in biomedical research, the National Institutes of Health (NIH) released a notice on June 9, 2015 entitled, *Consideration of Sex as a Biological Variable in NIH-funded Biomedical Research* that focuses on the “expectation that scientists will account for the possible role of sex as a biological variable in vertebrate animal and human studies” [5]. Furthermore, updated application instructions and review questions take effect for NIH applications submitted for the January 25, 2016 due date and thereafter. Greater understanding of the sex differences in incidence and progression of diseases will come from intensified study of the “sexome,” the sum of sex-biased effects on gene networks, and diverse cell and tissue systems [6].

To assist investigators in incorporating sex and gender into their research design and methodology, this review provides an annotated bibliography of currently available resources for conducting sex and gender research and analysis. The authors defined a resource as a broadly based tool that can be used to guide both novice and experienced researchers to consider the impact of sex and gender differences within research design and methodology. The authors believe that variables such as sex and gender are not monolithic variables but rather variables that interact and frequently reinforce each other. Examples of other variables that interact with biological sex include age, race, ethnicity, class, sexual orientation, and sexual identity. This review focuses on sex and gender as independent variables, as the first step in identifying their impact in medical research and health outcomes.

## Methods

The research team included seven academic researchers, both basic science and clinical science, from US universities. Together, they developed a protocol to guide the review process of material to be included in this resource. The review protocol included defining research questions, establishing the need for the review, inclusion and exclusion criteria, review period, timeline, and the resource selection/evaluation process.

The review was conducted to answer the following *research questions*:

1. What specific resources are available to assist students, researchers, educators, and policy makers with incorporation of sex and gender into health research design?
2. What is the format of the publication describing each resource? (journal article, review paper, report, webinar, book, case study, recorded didactic presentations)
3. To what extent are these resources freely available?
4. Who is the intended audience for these resources?
5. What is the major utility of each resource?
6. What is the level of “ease of use” for each resource?
7. What is the level of evidence on which the resources are based?

As a first step, we identified the need for a review of resources; an initial search of the literature did not reveal a review of resources on the topic. In 2014, the authors systematically searched published literature online (Medline using PubMed, Web of Science, Embase and Google Scholar for articles, text books, and other types of published resources) and by hand-search (bibliographies). The following search terms were used: sex analysis AND gender analysis AND tools for gender analysis AND research methods for sex and gender AND designing sex and gender analysis AND designing sex and gender research AND gender-based analysis. The period of review included articles published up to 2014 with no beginning date.

### Data collection and analysis

A “resources selection checklist” was developed to determine the eligibility of resources for inclusion in the review. Both basic and clinical science resources were included. Inclusion criteria required publication in the English language. The initial search yielded 69 resources. Each resource was examined to see if any additional citations could be located. From these searches, after eliminating resources that did not meet eligibility criteria, 38 resources were identified and a database of these resources was created.

There were a total of seven reviewers. The authors were divided into review teams, and each team had either two or three members. Each member of a review team independently assessed the identified resource and later compared and discussed their assessments with other team members to reach consensus. Disagreements were resolved through discussion or, if required, via adjudication by a third reviewer. Articles were excluded if, after review, they were not considered resources. A form was developed to extract data. Articles that were considered appropriate for inclusion after review team discussion were entered into a table using the data extraction form and reviewed in depth to answer the study research questions.

Table 1 presents the criteria used for data extraction. It should be noted that two of the criteria were later removed from final results “Audience” and “Level of Evidence”. The authors realized that the Audience could be broadly interpreted and many of the categories overlapped. The authors also discovered that there was insufficient data to determine the level of evidence for the resources.

**Table 1:** Data extraction criteria

Format	o Journal article about one resource o Journal article review of resource o Book o Power point o Webinar o Case study o Audio didactic o Audio-visual didactic
Availability	o Free online journal o University library o Internet access o Free e-book o Purchase e-book o Book most libraries would have o Book available in a university library o Book for purchase
Audience	o Basic researcher o Clinical researcher o Clinical practice o Medical student o Health professions students—not medical students o Residents o Policy o Public health
Level of evidence	Initial criterion: abandoned due to lack of evidence or not noted

## Results

### Data synthesis

After reviewing the final 38 resources, the research team divided them into five subcategories based upon similarities in research topic area. These were Basic Science; Clinical Research; Pharmaceuticals, Biologics, Pharmacokinetics, Devices; Epidemiology and Public Health; and Social and Cultural. The subcategories and the corresponding resources are presented in Table 2 and summarized below:

**Table 2:** Resources on conducting sex and gender Research

Subcategory	Resource title	Author	Resource type	Availability
1. Basic Science	Strategies and Methods for Research on Sex Differences in Brain and Behavior	Becker, J.B., et al.	Journal article	b, c
	Sex Differences in the Brain: The Not So Inconvenient Truth	McCarthy, M.M., et al.	Journal article	a, f
	Strategies and methods to study sex differences in cardiovascular structure and function: a guide for basic scientists	Miller, V.M., Kaplan, J.R., Schork, N.J. et al.	Journal article	a, c
	Integrating the dimensions of sex and gender into basic life sciences research: methodologic and ethical issues	Holdcroft, A	Journal article	c
	Sex bias in neuroscience and biomedical research	Beery, A.K. and Zucker, I.	Journal article	b, c
2. Clinical Research	Sex and Gender Subgroup Analyses of Randomized Trials: The Need to Proceed With Caution	Aulakh, A.K., et al.	Journal article	b, c
	Studying sex and gender differences in pain and analgesia: a consensus report	Greenspan, J.D., et al.	Journal article	b, c
	Sex and Gender in Systematic Reviews Planning Tool	Doull M., Runnels V., Tudiver S., Boscoe M.	Online tool (http://methods.cochrane.org/equity/sites/methods.cochrane.org.equity/files/uploads/SRTool_PlanningVersionSHORTFINAL.pdf)	c
	Better Science with Sex and Gender: A Primer for Health Research	Johnson, J.L., Greaves, L., & Repta, R.	Online tool (http://bccewh.bc.ca/wp-content/uploads/2012/05/2007_BetterSciencewithSexandGenderPrimerforHealthResearch.pdf)	c, d, e
	Thinking Critically About Research on Sex and Gender	PJ Caplan and JB Caplan	Book	f, g, h
	Designing and Conducting Gender, Sex & Health Research	Lindsay and Greaves	Book	b, e, f, g
	Appraising the evidence: applying sex- and gender-based analysis (SGBA) to Cochrane Systematic Reviews on cardiovascular diseases.	Doull, M et al.	Journal article	a, c
	Scientific excellence in applying sex- and gender-sensitive methods in biomedical and health research	Nieuwenhoven, L. and Klinge, I.	Journal article	b
	Gender Awakening Tool. Bibliography: Sex & Gender in Biomedical and Health Research	Gender Basic	Online tool (www.genderbasic.nl)	c
	The Hidden Science in Your Emergency Medicine Research: Gender-Specific Study Design and Analysis	Society for Academic Emergency Medicine	Online videos (https://vimeo.com/66486803)	c
	Sex and Gender Differences in Alzheimer’s Disease: Recommendations for Future Research	Carter, C.L., Resnick, E.M., Mallampalli, M., Kalbarczyk, A.	Journal article	b, c
	An interdisciplinary analysis of sex and gender in relation to the pathogenesis of bronchial asthma	Lux, R., Awa, W., Walter, U.	Journal article	b, c
	Sex Differences and Implications for Translational Neuroscience Research: Workshop Summary	IOM	Journal article	c, d, h
3. Pharmaceuticals, Biologics, Pharmacokinetics, Devices	Participation of Women and Sex Analyses in Late-Phase Clinical Trials of New Molecular Entity Drugs and Biologics Approved by the FDA in 2007-2009	Poon, R., Khanijow, K. et al.	Journal article	b
	Sex, Gender, and Pharmaceutical Politics: From Drug Development to Marketing	Fisher, J.A. et al.	Journal article	b, c
	De-Gendering the Knee: Overemphasizing Sex Differences as a Problem.	Gendered Innovations	Online tool (http://genderedinnovations.stanford.edu/case-studies/knee.html#tabs-1)	c
	How important are gender differences in pharmacokinetics?	Meibohm, B., Beierle, I., Derendorf, H.	Journal article	b
	Methodologic ramifications of paying attention to sex and gender differences in clinical research	M. Prins, et al.	Journal article	b
4. Epidemiology and Public Health	Sex and gender: the challenges for epidemiologists	Doyal, L.	Journal article	b
	Methodologic and Ethical Ramifications of Sex and Gender Differences in Public Health Research	Lawrence, K. and Rieder, A.	Journal article	b, c (not free)
	Rising to the Challenge: Sex and gender-based analysis for health planning, policy and research in Canada	Clow, B. and Pederson A., et al.	Online tool (http://www.pwhce.ca/pdf/RisingToTheChallenge.pdf)	b, d
	Women and Occupational Lung Disease: Sex Differences and Gender Influences on Research and Disease Outcomes	Camp, P.G., Dimich-Ward, H., Kennedy, S.M.	Journal article	b, c
	Genders, Sexes and Health: What are the Connections-and Why Does it Matter?	Krieger, N.	Journal article	b, c
	Neurotoxic exposures and effects: Gender and sex matter!	Mergler, D.	Journal article	b, c
5. Social and Cultural	Intersectionality: Moving Women’s Health Research and Policy Forward	Hankivsky, O. and Cornier, R.	Online tool (http://bccewh.bc.ca/wp-content/uploads/2012/05/2009_IntersectionaliyMovingwomenshealthresearchandpolicyforward.pdf)	c
	A Toolkit on Collecting Gender & Assets Data in Qualitative & Quantitative Program Evaluations	Gender, Agriculture, and Assests Project	Online tool (http://gaap.ifpri.info/files/2010/12/GAAP_Toolkit_Update_FINAL.pdf)	c
	A Tool for Developing Gender Research in Medicine: Examples from the Medical Literature on Work Life	Hammarström, A	Journal article	b, c
	Central Gender Theoretical Concepts in Health Research: The State of the Art	Hammarström, A., Johansson K., Annandale, E., Ahlgren, C., et al.	Journal article	b
	Doing gender in sex and sex research	Vanwesenbeeck, I	Journal article	a, b, c

^a^ Free online journal (free)

^b^ University library (UL)

^c^ Internet access (free)

^d^ Free e-book (free)

^e^ Purchase e-book (purchase)

^f^ Book most libraries would have (purchase)

^g^ Book available in a university library (UL)

^h^ Book for purchase (purchase)

#### Basic Science

In a systematic review entitled “Sex Bias in Neuroscience and Biomedical Research,” the authors found that for every single-sex study conducted in nonhuman female animals in 2009, there were significantly more conducted in males across eight out of ten biological disciplines. Remarkably, in the field of neuroscience the male-to-female ratio of single-sex studies was 5.5:1 [2]. The authors examined how the investigator preference for studying male animals originated, and they discuss the adverse consequences of this male sex bias in preclinical research for human health. This article is an excellent resource for gaining an understanding of the historic existence of a sex bias in preclinical animal and human research across ten major biological disciplines including Animal Behavior, Behavioral Physiology, Endocrinology, General Biology, Immunology, Neuroscience, Pharmacology, Physiology, Reproduction, and Zoology. One of the reasons preclinical research has historically been predominantly conducted on male animals is the mistaken assumption that female mammals are intrinsically more variable than males because of the estrous cycle. This assumption of greater female trait variability was called into question by Mogil and Chanda, who conducted a meta-analysis of nociceptive responses in forty strains of inbred mice and concluded that there were no sex differences in the response variability [7]. The article “Female mice liberated for inclusion in neuroscience and biomedical research” by Prendergast et al. is an excellent resource that extends these findings of Mogil and Chanda through a systematic review of the literature on the variability in behavioral, morphological, physiological, and molecular traits between male and female mice without consideration of the estrous cycle stage [8]. The authors found that for any endpoint studied, variability was not greater in females than males and in fact for several traits, the variability was greater in males.

“Sex differences in the Brain: The Not So Inconvenient Truth” is an excellent resource for understanding how to operationally categorize sex differences in endpoints [9]. The authors identify three classes of sex differences. Type I, classified “sexual dimorphism” is defined as an endpoint consisting of two forms. Included in type I differences are endpoints that are present in one sex but not the other. For example, only females get pregnant or, for behaviors, courtship rituals, and copulatory behavior differs between males and females. Type II, “sex differences” are defined as endpoints that exists on a continuum with the average or the variability around that average differing between the sexes as in pain sensitivity or olfactory recall ability. Type III, “sex convergence and divergence” defines endpoints that are the same in males and females but in which route to achieve the endpoint differs as in problem solving strategies in spatial learning tasks or endpoints that are identical at baseline but diverge in response to a particular challenge such as sex differences in vulnerability to environmental toxins. This paper is also a resource for how to study the cause of sex differences and includes decision tree strategies and the methodology for addressing these strategies. For example, the first recommended experiment is to determine if a sex difference is due to gonadal hormones since most reported sex differences are due to adult gonadal hormone levels. To address this question, the outcome parameter is compared between adult males and females in the intact and gonadectomized state. Then, depending upon the findings, gonadal hormone replacement studies may be indicated. While this article focuses on the brain, the concepts are relevant to many disciplines.

Another valuable resource for investigators interested in studying the causes of sex differences, not only in the brain but in also other fields as well, is “Strategies and Methods for Research on Sex Differences in Brain and Behavior” [10]. The authors organized this paper as a series of decision tree questions and then provided highly detailed methodologies on how to study the role of male and female gonadal hormones using endocrine ablation and hormone replacement therapy in animals and how to differentiate developmental from adult origin gonadal hormone effects. In addition, the authors discuss animal models that can be used to investigate the role of the sex chromosomes independently of the gonadal hormones and vice versa. Becker et al. offer detailed methods and caveats for measuring sex hormones in serum and saliva and the importance of taking into account the impact of stress in the study of sex differences of a particular trait. This paper also discusses how to gain insight into the cause of sex differences in humans by taking advantage of naturally occurring changes in the endocrine state across the male and female life span. Missing from this review, however, is the value of using naturally occurring endocrine disorders (e.g., polycystic ovary disease) and sex chromosome abnormalities (e.g., Turner Syndrome and Klinefelter syndrome) and studies of surgical or chemical clinical interventions (e.g., elective oophorectomies and super agonist-induced ablation of testosterone production).

Another excellent resource for investigators interested in how to design and conduct investigations into the cause of sex differences using cells and tissues from animals or whole animal physiology was written by Miller et.al. In addition to design considerations and choice of experimental models, in “Strategies and methods to study sex differences in cardiovascular structure and function: a guide for basic scientists”, the authors discuss the impact of the sex, environment, and history of the animal harvested for studies using animal tissues and cells with an emphasis on cardiovascular research; however, the approaches discussed are relevant for other fields as well [11]. This article highlights the need to consider reproductive status (e.g., pre- or post-pubertal, virgin, or numerous pregnancies) as well as the environment in which the animal lived including whether or not the animal was exposed to diets rich in phytoestrogens, experienced disrupted sleep/wake cycles, and/or subjected to social isolation or interaction. In addition, Miller et al. emphasize the importance of considering sex as a dichotomous variable as well as a covariate when both sexes are included within a study.

The use of the term “gender” in animal research remains controversial with some investigators preferring the word “sex” when referring to all studies of non-human male and female animals, while the Institute of Medicine [12] uses “gender” to define “the biology that is shaped by environment and experience”. Using the Institute of Medicine definition of “gender” in “Integrating the Dimensions of Sex and Gender into Basic Life Sciences Research: Methodologic and Ethical Issues” discusses the need to consider the impact of animal-to-animal interaction, i.e., the role of the physical and social environment in which animals are housed, as well as the role of the impact of the sex of the animal handler on the animal due to sex differences in human odors, sounds, and animal handling [13].

This paper is also an excellent reference for understanding the impact of how the accumulation of small sex differences can have major effects on outcomes. Methodological approaches to being able to recognize small sex differences by reducing experimental variation, sufficiently powering studies, and by conducting meta-analyses are presented. In addition, this article provides a valuable resource for considering overlooked variables in designing sex difference research including sex differences in the pharmacokinetics and pharmacodynamics of drug action when conducting a pharmacological intervention. This is an excellent resource for Animal Care and Use Committees during their review of animal protocols as Holdcroft recommends guidelines for encouraging disclosure of information that affects sex or gender differences such as strain variations and age of animals. Moreover, she provides the rationale for why preclinical research design should consider the impact of sex on disease incidence and outcomes.

#### Clinical Research

“Sex and Gender Subgroup Analyses of Randomized Trials” extends a vital message for researchers interested in conducting sex and gender subgroup analyses (SGA) of randomized clinical trials [14]. Investigators are cautioned that the possibility exists for misleading results for improperly conducted SGA, which could mistakenly influence medical management. Through a search of cardiovascular randomized clinical trials, the authors make the case that only 1/3 of them provided evidence of conducting a proper SGA. Guidelines for performing and interpreting a rigorous SGA are summarized in Table 3.

**Table 3:** Guidelines for conducting rigorous subgroup analysis by sex and gender in randomized controlled trials

1	State the subgroup analysis a priori
2	Provide a rationale for performing the subgroup analysis
3	Offer a hypothesis regarding the outcome of the subgroup analysis
4	Adjust *p* values for the number of comparisons being made
5	Emphasize overall findings instead of subgroup analysis findings because results can only be considered hypothesis generating

Adapted from “Sex and Gender Subgroup Analyses of Randomized Trials”

For a comprehensive primer to serve as a guide in conducting sex and gender-based analysis (SGBA) across the continuum of biomedical research, “Better Science with Sex and Gender: A Primer for Health Research” does just that [15]. This small book is accessible to those with even basic understanding of biology and outlines designs for SGBA within basic science and preclinical studies, clinical and healthcare systems research, and social and cultural determinants. Missing within its context are references concerning biomedical device, biomarker, and diagnostic test developments, which are important components of SGBA approaches.

The following resources for incorporating sex and gender into clinical research design and analysis referred to in publications as “Tools”:

- To plan health interventions or outcomes with the intent of performing a SGA, the appraisal tool, “Sex and Gender in Systematic Reviews Planning Tool”, assists researchers with the inclusion of appropriate questions regarding the possible impact of sex and gender [16]. The tool itself is easy to use and consists of six series of questions that include Background, Criteria for considering studies, Methods, Results and Analysis, Discussion and Conclusion, and Table of Included Studies.
- “Scientific Excellence in Applying Sex- and Gender-Sensitive Methods in Biomedical and Health Research” is the result of a workshop, “Scientific Excellence and ‘Sexy’ Research”, organized by the authors to develop methods of conducting sex and gender sensitive research and develop a tool that allows for detection of sex and gender bias throughout all phases of the research process [17]. The tool is a seven-step plan that reviews the main sex and gender issues to consider in each stage of research including (1) Relevance Check, (2) Literature Search, (3) Formulation of research questions and hypotheses, (4) Research methods and sample, (5) Data analysis and interpretation, (6) Reporting, and (7) Conclusions and recommendations. This resource is valuable for a broad range of scientists and offers an easy to use checklist designed to assist the integration of sex and gender aspects in biomedical ad health research methodologies.
- The “Gender Awakening Tool” is an online tool developed by the Center for Gender and Diversity at the Maastricht University in The Netherlands and adopted by the European Commission, with the aim of ensuring better integration of gender dimensions in basic life sciences research [18]. It provides an earlier version of the seven questions described above; however, it has limited utility as an actual tool and serves more in the capacity to promote and create awareness for consideration of sex and gender in research design.
- The Sex and Gender Appraisal Tool for Systematic Reviews (SGAT-SR) is described in “Appraising the Evidence: Applying Sex- and Gender-Based Analysis (SGBA) to Cochrane Systemic Reviews on Cardiovascular Diseases” [19]. The SGAT-SR was developed by the authors and is composed of 35 items which appraise the nine sections of a Cochrane review: (1) Background, (2) Objectives, (3) Criteria for inclusion/exclusion, (4) Search strategy, (5) Methods, (6) Analysis, (7) Discussion and Conclusions, (8) Quality assessment, and (9) Table of included studies [19]. This tool has not been externally validated but is systematic, detailed, and should be useful to assess appropriate application of SGBA to the evidence synthesized by systematic reviews across disciplines.
- Several articles address the evidence of sex differences in specific disciplines and disease processes “An Interdisciplinary Analysis of Sex and Gender in Relation to the Pathogenesis of Bronchial Asthma,” “Sex and Gender Differences in Alzheimer’s Disease: Recommendations for Future Research,” and “Studying Sex and Gender Differences in Pain and Analgesia: A Consensus Report” [20]. These are invaluable resources to assist researchers in identifying sex-based research questions for future areas of study in particular medical specialties. “Sex Differences and Implications for Translational Neuroscience Research: Workshop Summary” is an extensive report approved by the Governing Board of the U.S. National Research Council (2011) with recommendations for studying sex differences in medicine, translational research, drug development, and reporting these differences in research publications [21]. This broader viewpoint enables this resource to be a valuable template for workshops addressing sex differences in many disciplines.
- For those inclined to watch video presentations, The Society for Academic Emergency Medicine hosted a didactic presentation moderated by Choo, EK, called “The Hidden Science in Your Emergency Medicine Research: Gender-Specific Study Design and Analysis” [22]. This symposium covers examples of sex differences in outcome and diagnostics using traumatic brain injury and cardiovascular research as examples, which serves as a useful motivator for researchers to explore sex differences in clinical medicine. There is also a focus on statistical design of studies assessing the impact of sex and gender.
- Additionally, two books were identified as clinical research resources. The first, “Thinking Critically About Research on Sex and Gender” provides one of the earliest sources of guidance in conducting sex and gender research as it was copyrighted in 1994 [23]. Included in the book is a history of the science of sex differences, a description of the scientific method, and particular chapters on what were current issues at the time, including math, spatial ability, women and masochism, males and aggression, mother-blame, women and hormones, verbal ability, and dependence of females. Its limitations lie in the fact that it is over 20 years old and the information is mainly historical. However, surprisingly, it is still relevant. “Designing and Conducting Gender, Sex, & Health Research” thoroughly examines study concepts, design, measurement, qualitative analysis, and public health policies divided into three parts as areas of considerations for gender and sex research [24]. A collective of chapter authors from the Canadian Fulbright Regional Network for Applied Research (NEXUS) Program contributed to this comprehensive resource.

#### Pharmaceuticals, Biologics, Pharmacokinetics, and Devices

“Sex, Gender, and Pharmaceutical Politics: From Drug Development to Marketing” provides an overview of the drug development process [25]. This resource is helpful to researchers to understand US and international drug regulation and marketing as well as clinical drug development. Cases of both underinclusion and overinclusion of women in clinical drug development programs are presented and associated efficacy and safety concerns are discussed. “Participation of Women and Sex Analyses in Late-Phase Clinical Trial” presents a method to assess participation by women in late phase clinical trials of drugs and biologics [26]. Ratios were calculated according to the proportion of women in the disease population. Presentation of the ratio for new drugs by disease and for biologics by disease is presented for multiple diseases. This analysis can assess the adequacy of representation by female subjects in clinical trials.

While the goal was to report on sex and gender differences in pharmacogenetic studies, “Methodologic Ramifications of Paying Attention to Sex and Gender Differences in Clinical Research” is a resource for reporting sex and gender in clinical research in general [27]. Included is a table with a helpful list of questions to ask about study design and analysis when thinking about sex and gender issues in clinical research. “How Important are Gender Differences in Pharmacokinetics?” reviews mechanisms relevant to drug absorption and distribution that have been shown to exert gender-specific activity differences [28]. This well-organized article could provide a path to investigate gender differences in pharmacokinetics for a new drug, but it should be noted that pregnant women and women on hormonal therapies were excluded from this review.

In “DeGendering the Knee: Overemphasizing Sex Differences as a Problem,” a medical marketing case study is presented [29]. This case puts forward the idea that sex differences can be overemphasized to the exclusion of other factors, such as height and weight. Thus, sex differences can be emphasized for reasons having more to do with medical marketing than optimal patient care.

#### Epidemiology and Public Health

“Sex and Gender: The Challenges for Epidemiologists” is an excellent piece that acknowledges limitations in epidemiologic and statistical analysis in performing and declaring research designed to depict sex and gender differences [30]. It begins by defining sex and gender and underscores the importance of sex and gender research. The authors further outline challenges in determining accurate differences attributable to sex and gender, from an epidemiologists’ perspective, and reviews strategies for mainstreaming sex and gender into health research.

“Methodologic and Ethical Ramifications of Sex and Gender Differences in Public Health Research” is a review of articles that examine the current status of gender in public health research [31]. The review thoroughly discusses ethical barriers to public health studies. It answers as well as discusses questions on gender biases, ethics, and methodologies, and the establishment of guidelines and offers recommendations for improving gender representation and evaluation on ethics committees and in public health research methodology (including that data are disaggregated by sex and by socioeconomic factors).

“Rising to the Challenge: Sex and gender-based analysis for health planning, policy and research in Canada” is an in-depth source of information about sex/gender and processes that recognize the effects of sex and gender in research, policy, programs, etc. [32]. This resource provides background on the origins of sex- and gender-based analysis and the major concepts of sex, gender, diversity, and equity as well as how these concepts relate to health. It also provides guidance on how to conduct sex- and gender-based analysis. This piece stresses the importance of assessing the impact of other variables (e.g., age, ethnicity, race, and socio economic status) on health, and provides case studies illustrating the power of sex and gender bases analyses for understanding different types of health, health care, and health policy issues. It complements introductory guides and checklists by inviting readers to engage in a deeper, extended discussion about the changing meanings of “sex” and “gender” and their current and potential roles in health and society. It introduces the concept that more complex analyses are key to moving beyond simple assessments of differences between women and men toward an understanding of why these differences exist and how best to respond to them. Finally, this resource treats sex- and gender-based analysis as a process, rather than a tool or template, thereby emphasizing its flexibility and transferability across sectors, disciplines, and regions.

“Women and Occupational Lung Disease: Sex Differences and Gender Influences on Research and Disease Outcomes” is a journal article review that is readily accessible to those with a general understanding of human subject research [33]. This article describes the limitations of what is known regarding the role of gender in occupational lung disease. The authors focus attention on the challenges faced by researchers when investigating the impact of gender in occupational health and disease, which includes sex and gender differences in tasks, work shifts, effectiveness of protective practices and measures, lung mechanics, co-morbidities, and pre-existing conditions as well as the limitations in record keeping by sex. The authors do not separate the concepts of sex and gender in their review; identified gaps in knowledge include the lack of specific data being collected by sex and/or gender such as symptom reporting and the potential role of gender bias in reporting. This resource serves as an example of the methodological challenges that face researchers when investigating the influence of sex and gender in occupational-associated diseases including differences in perception, and the interaction of environmental, sociocultural, and biologic factors.

“Genders, Sexes and Health: What are the Connections-and Why Does it Matter?” is a commentary and analysis that summarizes the conceptual debates leading to distinctions between “sex” and “gender” as biological and social constructs [34]. The resource can be used as a guide in integrating gender mainstreaming at every stage of research. This article educates scientific researchers in the complexity and multiple ways the factors of sex and gender can independently and synergistically affect health outcomes. The case examples presented in the paper highlight that gender relations influence expression—and interpretation—of biological traits, and also that sex-linked biological characteristics can, in some cases, contribute to or amplify gender differentials in health.

“Neurotoxic exposures and effects: Gender and sex matter!” is a well-organized review article and tool that summarizes evidence on sex and gender differences in neurotoxic exposure and provide suggestions for sex- and gender-sensitive research in neurotoxicology [35]. The author breaks down suggestions to apply to each step of the research process, from developing research questions and study design to conducting the study, analyzing and interpreting findings, to publication of findings.

This resource will be most useful to researchers, scientific journal editors, and grant funders.

#### Social and Cultural

“Central Gender Theoretical Concepts in Health Research: The State of the Art” clarifies key concepts and language used in sex and gender research critical to research design and manuscript development [36]. It highlights the importance of classifying gender theoretical concepts as central and interlinked in health sciences through definitions of sex, gender, intersectionality, embodiment, gender equity, and gender equality. “Doing gender in sex and sex research. Archives of Sexual Behavior” is a discussion of gender versus sex with gendered sexuality as a social process [37]. This particular essay discusses ten difficulties in the treatment of gender in sex research, reflects on their origins, and reviews the theory behind it. This resource may be useful to researchers as a basic platform for gender-based studies.

“Moving Women’s Health Research and Policy Forward” addresses the critical concept of “Intersectionality” [38]. Women’s health and experiences are shaped not only by sex and gender but also by other factors such as race, class, culture, income, education, age, ability, sexual orientation, immigration status, ethnicity, indigeneity, geography, and so on. The purpose of this primer is to explore the following question: How can health researchers, policy analysts, program and/service managers, decision makers, and academics effectively apply an intersectional perspective to their day-to-day work? The primer includes the following: an overview of intersectionality including challenges and advantages of this approach; a discussion of the key assumptions of intersectionality; comparison and contrast of an intersectional approach, a gender- or sex-based approach, a health determinants approach, community-based research, and indigenous approaches; a discussion of the need for an intersectional approach in gender and women’s health research; discussion of how to integrate an intersectional approach into health research; a discussion of how to integrate an intersectional approach into health policymaking; and examples of the application of an intersectional framework to three health issues.

“A Toolkit on Collecting Gender & Assets Data in Qualitative & Quantitative Program Evaluations” is a toolkit devised to assist data collection on household assets for research on development programs [39]. Previous research in this area used pooled data from both genders; where in actuality men and women have and use their household assets differently. This toolkit provides definitions, statistical methods, and case studies designed to assist the researcher in collecting appropriate gender data. This online toolkit is organized in three sections. The first section provides an overview of the key concepts in gender and asset ownership. The second section describes measures for assessing the impact of gender on asset data collection including the tools, best practices, and approaches as well as the limitations of these methods. The final section summarizes the best practices and provides key recommendations for gender asset collection. An appendix offers additional resources, case studies, and a guide for integrating gender into household surveys.

“A Tool for Developing Gender Research in Medicine: Examples from the Medical Literature on Work Life” [40] is a journal article that has two objectives. One is to perform a review on work-life research to determine the number of articles that take sex/gender into account. The second is to develop a model to summarize characteristics that can differentiate sex and gender blind research, sex and gender differences research, and gender research. The goal is to encourage researchers to become aware of shortcomings in traditional gender-blind research and the developmental potentials in gender research.

The model is referred to as a “Tool”; however, it is more accurate to refer to it as a table that defines characteristics of research studies that are sex/gender blind versus sex/gender differences versus gender research. The limitation of this model is that the complexities of the connections between sex and gender are not fully taken into account. This resource can be used in assisting researchers in defining their current research as sex/gender blind versus gender research as well as encourage the design of research that takes sex/gender into account. This paper can be used by investigators reviewing the literature for studies on the impact of gender in human health, and it provides a model for distinguishing among sex/gender blind research, sex/gender difference research, and gender research.

## Conclusions

It is critically important to consider both sex and gender in research design, implementation, analysis, and interpretation. Sex- and gender-based comparisons can provide clues into disease mechanisms that could lead to new drug targets and treatment strategies, while analyzing data by sex and gender will enhance scientific rigor and reproducibility. Not considering sex and gender has adverse consequences for human health, not only through missed opportunities for therapeutic discovery but because sex and gender can impact the frequency and magnitude of adverse events. NIH has recognized the importance of these issues and, soon, failure to take sex into account will make applicants noncompliant when applying for NIH funding. Notice NOT-OD-15-102 released by NIH on June 9, 2015 requires that all applications submitted to the NIH on January 25, 2016 and thereafter, “NIH expects that sex as a biological variable will be factored into research designs, analyses, and reporting in vertebrate animal and human studies. Strong justification from the scientific literature, preliminary data, or other relevant considerations must be provided for applications proposing to study only one sex” [5]. Including sex as a biological variable into research design can be a new and potentially complicated process for both established researchers that now need to change the way in which they have been conducting research and for new researchers seeking guidance in developing appropriate approaches.

This timely review provides a state-of-the-art annotated bibliography of sex and gender analytic resources to encourage and assist researchers in their efforts to design studies that include sex and gender, and to collect, analyze, and report sex and gender disaggregated data. Additionally, the authors sought to perform a review of each resource identified using previously established qualities. To our knowledge, such a comprehensive review has not previously been conducted.

Our review of the literature through 2014 yielded 69 resources. After excluding resources that did not meet the eligibility criteria, 38 resources were identified and a database of these resources was created. Of the seven questions the review team set out to answer, we were not able to answer two questions. For question 7: “What is the level of evidence on which the resources are based?” insufficient data was available to determine the level of evidence for the identified resources. This lack of data could potentially represent a lack of rigorous study design for the resources and may indicate limitations in their future impact and reproducibility. In addition, this highlights the continued need to improve available resource tools for researchers that meet rigorous standards and measured outcomes. Additionally question 4: “Who are the intended audience of these resources?” was excluded as the “audience” could be broadly interpreted and many of the audience categories overlapped.

Taking both sex and gender into account in research are necessary, although not sufficient, to create evidence-based prevention, screening, diagnosis, and treatment plans for individuals and populations. There are multiple dimensions to sex and gender, which are important to consider—such as race/ethnicity, class/caste, age but are beyond the scope of this review.

Since the completion of this study, several resources have become newly available to assist researchers. A key resource is the NIH Office of Research on Women’s Health “The Science of Sex and Gender in Human Health,” an online educational source for courses offered as continuing education at no cost. Future resource tools such as this will be crucial to the successful integration of the new NIH mandate. Additionally, the landmark textbook that gathers important information in the field of sex- and gender-based biology and clinical medicine, “Principles of Gender-Specific Medicine: Gender in the Genomic Era” by Legato will soon be available in its third edition. Repositories for these resources will also be imperative as knowledge and access to varied resources is currently challenged by the lack of a centralized institute. The Sex and Gender Women’s Health Collaborative (SGWHC) is an interdisciplinary group of clinicians and researchers that through a collaborative effort created a digital library of evidence-based sex and gender resources to foster a sex and gender approach to research, education, and health. The authors anticipate that this review will also serve to broaden the outreach of SGWHC and similar organizations that serve to provide channels for sex- and gender-based health scientific journal discovery.

Based on our review and analysis, we propose the following recommendations to assist and guide research development and dissemination of the incorporation of sex and gender in research.

1. Funding agencies should assign preference points to grant proposals which include a sex and gender component in the study design, data collection, data analysis, interpretation, and reporting. An example would be the mechanism used by Health Resource and Services Administration grants for a variety of statutory funding preferences.
2. Institutional Review Boards for ethical conduct of human subject research and the FDA should make it mandatory for researchers to take into account sex and gender in the design and conduct of new research studies.
3. Institutional Offices of Research Development should assist investigators in developing their research projects with a sex and gender lens.
4. Scientific journals should consider expanding publication criteria for new manuscript submissions—particularly for papers focused on evidence-based medicine—to ensure that sex and gender are addressed

Limitations of this review are the consideration that resources are continuing to be developed and released that may not have been included by the time of publication. This is a consequence of the rapidly emerging recognition of the incorporation of sex and gender in research design. The review also did not include languages other than English.

This review of resources provides much needed information to assist basic researchers, clinical investigators, epidemiologists, population, and social scientists in considering the impact of sex and gender in their field of investigation and encourage the design of studies to elucidate the cause and consequences of these fundamental variables. There is a growing appreciation in the biomedical community of (1) the value of studying the role of sex and gender in measures and outcomes and (2) of how sex- and gender-based comparisons can inform research into disease mechanisms and the development of new therapeutics. Analyzing data by sex and gender will also improve the rigor and reproducibility of science. We expect that this review will facilitate individual selection of helpful resources and tools to expand toolboxes and widely assist a diverse audience of biomedical researchers to conduct research that ultimately aims to improve health outcomes for men and women.
